# *Pasteurella multocida* line infection: a case report and review of literature

**DOI:** 10.1186/s12879-018-3329-9

**Published:** 2018-08-23

**Authors:** T. C. S. Martin, J. Abdelmalek, B. Yee, S. Lavergne, M. Ritter

**Affiliations:** 1 Division of infectious diseases and global public health, University of California San Diego, San Diego, USA; 2 Division of nephrology, University of California San Diego, San Diego, USA; 3 Division of medicine, University of California San Diego, San Diego, USA

**Keywords:** *Pasteurella multocida*, Line infection, Bacteremia

## Abstract

**Background:**

There are as many as 300,000 visits to the emergency department in the USA with animal bites every year. The most common infection after cat or dog bite is with *Pasteurella Multocida*. Many people infected will also have long-term central venous access for dialysis or for other reasons. No prior reports or guidelines exist regarding the management of *P. multocida* bacteremia due to line infection or bacteremia in the presence of long-term central venous access. We describe the successful treatment of an individual with *P. multocida* bacteremia secondary to tunnelled line infection managed with line retention.

**Case presentation:**

A 21 year-old man with a history of granulomatosis with polyangiitis on home hemodialysis presented with fever and hypotension 3 days after dialysis catheter replacement. The patient was found to be bacteremic with *Pasteurella Multocida* and he subsequently reported a history of cat bite to his dialysis catheter*.* He declined removal of the tunnelled catheter and was thereafter treated for a total of 2 weeks with intravenous ceftazidime post-dialysis and gentamicin line-locks without recurrence of infection.

**Conclusions:**

*Pasteurella Multocida* bacteremia in the presence of a long-term central venous catheter is potentially curable using 2 weeks of intravenous antibiotics and line retention. Further data regarding outcomes of treatment in this setting are required though in select cases clinicians faced with a similar scenario could opt for trial of intravenous therapy and retention of central venous catheter.

## Background

Long-term central venous access is common in the developed world among patients on dialysis, individuals undergoing chemotherapy and people who are on long-term antibiotics among others. Simultaneously, animal bites are common accounting for approximately 1% of all emergency room visits annually in the USA thus suggesting that infection with zoonotic organisms, in the setting of a long-term catheter, is a problem that arises not infrequently [1]. Following both cat and dog bites the most common infection is with *Pasteurella multocida*, a gram-negative, facultative anaerobic non-spore-forming coccobacilli that can be seen in pairs or short chains. The optimal treatment of *P. multocida* bacteremia in the setting of a long-term central venous catheter or caused by infection of a central venous catheter is unknown. Existing guidelines published by the Infectious Diseases Society of America suggest that gram-negative bacterial infections of central venous catheters may be treated with 10–14 days of targeted intravenous therapy though no prior cases caused by *P. multocida* have been described in the literature [2]. We report the successful treatment of an individual with *P. multocida* bacteremia secondary to line infection with dialysis line retention.

## Case presentation

A 21 year-old man with a history of granulomatosis with polyangiitis on home haemodialysis presented to the emergency department with 12 h of fatigue, several episodes of watery diarrhea and nausea but no emesis. He reported chills, diaphoresis, light-headedness and fever as well as tenderness over his tunnelled catheter side. He suffered from chronic arthralgia in his knees. Otherwise he denied cough, sputum production or haemoptysis, sore throat, abdominal pain, headache or rashes. His tunnelled catheter had been replaced over a wire 3 days prior due to a small hole in one of the ports. The patient reported that the tenderness over the catheter site was usual after replacement.

His previous medical history included granulomatosis with polyangiitis causing end-stage renal disease, subglottic stenosis, pericarditis, central nervous system vasculitis, pulmonary haemorrhage, atrial thrombus and deep vein thrombosis. He also reported depression and low testosterone. He had undergone two previous renal transplants, the most recent 6 months prior to presentation for which he had received tocilizumab, alemtuzumab, mycofenolate mofetil, tacrolimus and prednisone. Both transplants were complicated by acute rejection and both native and graft nephrectomies had been performed. He was now established on nightly home hemodialysis five times weekly via a tunnelled left internal jugular catheter. He denied smoking, alcohol or drug use. Medications prior to admission included topical gentamicin ointment around the catheter entry site and he reported previously developing an itchy rash with penicillins.

The patient appeared chronically unwell with mild distress. His pulse was 135 beats per minute, blood pressure 82/39 mmHg, oxygen saturations via finger probe 95% on room air with a temperature of 103.2 F. Examination was notable for tenderness over the tunnelled catheter site without overlying erythema or purulent drainage. Jugular venous pressure was estimated at 6 cm of water.

Laboratory results on admission were notable for white cell count of 3400/mm3. Chest radiograph showed no evidence of acute cardiopulmonary disease and a tunnelled left internal jugular catheter terminating over the right atrium.

**Fig. 1:**
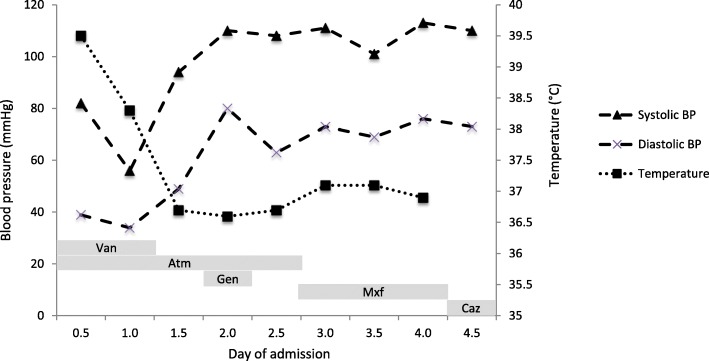
Chart showing temperature, systolic and diastolic pressures during admission. Grey boxes indicate the sequence of antibiotics administered during the inpatient period (Vancomycin (Van), Aztreonam (Atm), Gentamicin (Gen), Moxifloxacin (MXF), Ceftazidime (Caz))

The patient was admitted to the intensive care unit and placed empirically on vancomycin and aztreonam due to his penicillin allergy. A single dose of gentamicin 3 mg/kg was administered after gram-negative rods were identified in the blood cultures and cautious fluid boluses were given. The patient declined central line or arterial line placement. His midodrine was increased to 10 mg three times daily and he was started on norepinephrine via peripheral intravenous catheter. His haemodynamic parameters, fever chart and antibiotics administered are shown in Fig. 1. After 12 h, the patient was alert and oriented, his hands and feet were warm and lactate 0.5 mmol/L. Norepinephrine was discontinued. Initial blood cultures drawn in the emergency department were reported as positive with gram-negative rods growing in the aerobic and anaerobic bottles with time to positivity of 15 h. Speciation was performed using matrix assisted laser desorption/ionization –time of flight (MALDI-TOF) revealing *Pasteurella multocida* sensitive to erythromycin (minimum inhibitory concentration (MIC) 4mcg/mL), moxifloxacin (MIC 0.023 mcg/mL), penicillin G (MIC 0.064 mcg/mL) and tetracycline (0.75 mcg/mL). Peripheral and central venous catheter blood cultures drawn 12 h after admission were negative.

Further history from the patient revealed that the patient’s kittens played with his tunnelled catheter with a bite leading to the small puncture in one of the ports prior to admission. He was initially switched to intravenous moxifloxacin after susceptibilities were known then ultimately ceftazidime 1 g after dialysis 5 times weekly with gentamicin catheter locks. The choice of ceftazidime was based on ease of dosing with dialysis. He was discharged on day 4. He completed 2 weeks of antibiotics with surveillance blood cultures drawn 5 and 13 days after completion remaining negative.

## Discussion

We describe a case of *Pasteurella multocida* bacteremia in a patient that was dependent on a tunnelled catheter for dialysis and document the successful treatment with line retention and 2 weeks of intravenous antibiotics administered with dialysis.

*P. multocida* infection is common in particular after cat bite due to the depth of puncture caused by cats’ teeth. Infection can result in a number of complications including necrotising fasciitis, septic arthritis, osteomyelitis and less commonly septic shock, endocarditis and meningitis. The bacteria have several potential virulence factors including capsular lipopolysaccharide, a cytotoxin, hemagglutin, adhesins and iron acquisition proteins [3]. More severe disease has been documented in infants, pregnant women, patients on steroids, people living with HIV and immunocompromised patients [4]. In general, the organism is very sensitive to penicillin though some β-lactamase-producing isolates have been reported and empiric treatment is recommended with β–lactam/β–lactamase combinations such as ampicillin/sulbactam or amoxicillin/clavulanic [5]. Alternatives that have shown good activity against *P multocida* include second and third generation cephalosporins, tetracyclines, co-trimoxazole and fluoroquinolones.

Infection of prosthetic material with *P. multocida* following animal bites or licks is rare but has been described for prosthetic joints, aortic endografts and peritoneal catheters [6–8]. One retrospective study in France identified six *P. multocida* cases among 4686 prosthetic joint infections. All patients underwent surgery with prosthesis retention in three. Long-term antibiotics were used for all patients (range 6–18 months) without relapse at least 3 years after end of treatment [6]. The authors reviewed the literature identifying an additional 26 cases of *P. multocida* prosthetic joint infection. About half of these were treated with prosthesis retention (with or without debridement) with only four requiring subsequent prosthesis removal and staged replacement. An earlier study described aortic endovascular graft infection following rabbit bite and reviewed another two cases of endograft infection. The authors concluded in these cases that operative management is optimal with removal of infected material [7].

Perhaps most relevant to our case is a study of *P. multocida* peritonitis associated with peritoneal dialysis catheters [8]. The study reviewed seven local cases and 30 previously published reports with the authors concluding that although most patients were treated with catheter retention (89%), relapse occurred in only one patient. Despite significant heterogeneity in treatment the authors suggested that a 14-day course of intraperitoneal antibiotics was most likely adequate. They recommended use of penicillin or ampicillin-based regimens for non-β-lactamase producing isolates. Alternatives included third generation cephalosporins, ceftazidime and oral fluoroquinolones. One of three patients treated with intraperitoneal aminoglycosides had recurrence of infection thus the authors recommend against monotherapy with aminoglycosides.

In our patient, the infection route was via direct inoculation and line replacement was performed for punctured tubing. Bacteraemia ensued but was successfully treated without relapse, despite line retention, with a 14-day course of susceptible antibiotics intravenously and with catheter locks. Our case suggests that in cases of *P. multocida* infection with bacteremia, individuals can be considered for a trial of treatment with IV antibiotics, catheter locks and line retention. This is supported by the literature of successful treatment with prosthesis retention for prosthetic joint infections and peritoneal catheter associated infections. Given the discussed evidence for treatment failure of intraperitoneal aminoglycosides, the authors recommend catheter lock with either ampicillin or a cephalosporin such as ceftazidime depending on isolate sensitivities. In the case of purulent line infection, we believe it would be prudent to advocate for line replacement as recommended in Infectious Diseases Society of America (IDSA) guidelines [2].

To our knowledge this is the first description of a line associated *Pasteurella multocida* infection and provides management guidance for clinicians managing similar infections. Clearly recommendations are limited by the paucity of evidence; however, we believe that such infections are likely more common than reported in the literature and clinicians will benefit from our experience.

## Abbreviations

IDSA: Infectious Diseases Society of America
MALDI-TOF: Matrix assisted laser desorption/ionization –time of flight
MIC: Minimum inhibitory concentration

## References

[1] Oehler RL, Velez AP, Mizrachi M, Lamarche J, Gompf S. Bite-related and septic syndromes caused by cats and dogs. Lancet Infect Dis. 9:439–47. 10.1016/S1473-3099(09)70110-0 19555903

[2] Mermel LA, Allon M, Bouza E, Craven DE, Flynn P, O'Grady NP, Raad II, BJA R, Sherertz RJ, Warren DK (2009). Clinical practice guidelines for the diagnosis and Management of Intravascular Catheter-Related Infection: 2009 update by the Infectious Diseases Society of America. Clin Infect Dis.

[3] Wilson BA, Ho M (2013). Pasteurella multocida: from Zoonosis to Cellular Microbiology. Clin Microbiol Rev.

[4] Weber D, Wolfson J, Swartz M, Hooper D (1984). Pasteurella multocida infections. Report of 34 cases and review of the literature. Medicine (Baltimore).

[5] Christenson ES, Ahmed HM, Durand CM. Pasteurella multocida infection in solid organ transplantation. Lancet Infect Dis. 15:235–40. 10.1016/S1473-3099(14)70895-3 25467649

[6] Honnorat E, Seng P, Savini H, Pinelli P, Simon F, Stein A (2016). Prosthetic joint infection caused by *Pasteurella multocida:* a case series and review of the literature. BMC Infect Dis.

[7] Silberfein EJ, Lin PH, Bush RL, Zhou W, Lumsden AB (2006). Aortic endograft infection due to Pasteurella multocida following a rabbit bite. J Vasc Surg.

[8] Poliquin PG, Lagacé-Wiens P, Verrelli M, Allen DW, Embil JM (2015). Pasteurella species peritoneal dialysis-associated peritonitis: household pets as a risk factor. Can J Infect Dis Med Microbiol.

